# CLINICAL PROFILES AND OUTCOMES OF ADMISSIONS FOLLOWING COVID-19 ADMISSIONS DURING THREE WAVES OF THE PANDEMIC: EXPERIENCE OF A TERTIARY HOSPITAL IN THE EASTERN CAPE PROVINCE OF SOUTH AFRICA

**DOI:** 10.21010/Ajidv18i2S.3

**Published:** 2024-07-04

**Authors:** KABAMBI Kasandji Freddy, JOSEPH Sibi Sebastian, PANICKER Mahesh Kumar, NDLOVU Nomagugu, EKPEBEGH Chukwuma

**Affiliations:** 1 Department of Internal Medicine, Walter Sisulu University, P Bag x1, UNITRA 5117, Mthatha, South Africa; 2 Cardiometabolic research Niche, Walter Sisulu University, P Bag X1, UNITRA 5117, Mthatha, South Africa

**Keywords:** Covid-19, Eastern Cape, South Africa, Mortality, Hypertension, Diabetes, HIV infection

## Abstract

**Background:**

South Africa was the country worst affected by the Covid-19 pandemic in Africa. There is a paucity of data on the clinical characteristics and mortality of Covid-19 from the Eastern Cape province of South Africa. We report on the demographic and clinical characteristics as well as the mortality of patients admitted to the Covid-19 ward of Nelson Mandela Academic Hospital (NMAH), Mthatha, during three waves of the Covid-19 pandemic in South Africa.

**Materials and Methods:**

We conducted a single centre retrospective observational study of patients admitted for Covid-19 in a tertiary hospital in the rural Eastern Cape of South Africa. Data were collected from patient files, electronic databases and the National Health Laboratory Services (NHLS) database. The outcomes were duration of admission and in-hospital mortality.

**Results:**

There were 371 patients admitted across all three waves with a mean age of 52.2 ± 16.3 years. The proportion of females across the three waves is 61.2%. The commonly associated comorbidities, irrespective of the wave, were hypertension, diabetes and HIV infection. The median duration of admission was six days, with an overall mortality of 31%. The mortality for first, second and third wave were 29.3%, 31.5% and 37.9% respectively.

**Conclusion:**

Admissions for Covid-19 were predominantly in females and middle-aged. One third of the admitted patients died. Diabetes, hypertension and HIV infection were the most commonly associated comorbidities.

## Introduction

Covid-19 caused by the SARS-COV-2 virus, was first reported in Wuhan, Hubei province of China, in December 2019 and declared a pandemic by the World Health Organization in March 2020. South Africa has reported the highest number of Covid-19 infections and deaths in the African continent (Cabore *et al.*, 2022).

There have been three waves of Covid-19 in South Africa. These three waves have occurred during different circumstances in terms of viral variant, season, institutional preparedness, treatment modalities and availability of vaccines. The first, second and third waves have been dominated by the beta, delta and omicron variants of the SARS-COV-2 virus, respectively (Mabuka *et al.*, 2023). The first and third waves occurred in winter, while the second wave was in summer. There was greater institutional preparedness and experience for the second and third waves than for the first. There was also a more uniform treatment approach by the attending medical practitioners guided by an institutional treatment protocol that was available and widely circulated before the third wave admissions (Maison *et al.*, 2021). Furthermore, the national vaccination program, which was non-existent before the first and second waves, had commenced for healthcare workers and people older than 60 years of age before the third wave of Covid-19 infection with the potential dual effect of protecting frontline healthcare workers and elderly patients who are at the greatest risk for adverse Covid-19 disease outcomes (Piltch-Loeb *et al.*, 2023).

The Nelson Mandela Academic Hospital, Mthatha, in the Eastern Cape province of South Africa, is a tertiary hospital serving about 3 million people. In the wake of the Covid-19 pandemic, a 30-bed facility was established to manage Covid-19-positive patients requiring hospitalisation. Treatment of Covid-19 in Nelson Mandela Central Hospital, Mthatha has varied during the three waves of Covid-19 infection. In the first wave, from May 2020 to August 2020, treatment included chloroquine, ritonavir-boosted lopinavir and remdesivir as part of the World Health Organization (WHO) sponsored multinational and multi-centre solidarity clinical trial (Pan *et al.*, 2021).

There were concerns with the use of steroids in the first wave as steroids may induce immune suppression with the potential for increased viraemia and the occurrence of secondary bacterial and fungal infections. In the second wave, from December 2020 to April 2021, steroids became the standard of care for our patients needing oxygen therapy following the results from the recovery trial (Horby *et al.*, 2021). The protocol for the treatment of the 3rd wave, which lasted from July 2021 to September 2021, excluded the use of chloroquine, lopinavir-ritonavir and remdesivir following results of no mortality benefit from these drugs (Pan *et al.*, 2021). Our current protocol includes prophylactic or therapeutic enoxaparin depending on D-Dimer level, steroids for oxygen-requiring patients (10 days course of parenteral dexamethasone at 8 mg daily or a 3-day course of methyl prednisone at 250 mg daily followed by parenteral dexamethasone at 8 mg daily or oral prednisolone at 40 mg daily) and insulin (twice daily subcutaneous protophane and sliding scale subcutaneous actrapid) therapy for hyperglycaemia. Patients also received 50,000 units stat dose of calciferol, 20mg of Zinc and 1000 mg of vitamin C daily. Patients with diabetic ketoacidosis received actrapid infusion intravenously. Antibiotics (7 days course of intravenous ceftriaxone and a 5-day course of azithromycin) were given for suspected bacterial infection. Patients with Acute Kidney Injury unresponsive to fluids therapy underwent haemodialysis in the Covid-19 ward. Medications for existing comorbidities were usually continued except where they were contra-indicated in severe covid-19 disease. This study reports the clinical profiles and outcomes of admissions for Covid-19 during three waves of the pandemic at the Nelson Mandela Central Hospital, Mthatha, Eastern Cape province of South Africa.

## Materials and Methods

### Study Design

Retrospective, cross-sectional review of patients’ records admitted at Nelson Mandela Academic Hospital, Mthatha, Eastern Cape province of South Africa. This study was approved by the Walter Sisulu University Faculty of Health Science Human Research Committee (protocol number 126/2021).

### Study setting

Nelson Mandela Central Hospital is a tertiary referral centre in the O.R Tambo District of Eastern Cape, South Africa. There was a dedicated Covid-19 ward at the hospital with 30 beds, 4 high care/Intensive care unit beds with a ventilator per bed, and a dedicated X-ray and dialysis machine.

### Study participants

These were patients with a confirmed diagnosis of Covid-19. In the first wave, patients were admitted regardless of the severity and discharged when PCR was negative. In subsequent waves, only those that required oxygen therapy or other comorbidity that required in-patient care were admitted into the ward.

### Data collection

Using a standardised data collection form; we collected data from the patient files and the National Health Laboratory Service (NHLS) database. The data collected included demographic variables, comorbidities, clinical characteristics, laboratory parameters, treatment received, length of hospital stay and mortality.

## Statistical analysis

We reported the baseline characteristics using descriptive statistics. Categorical data are reported as count (percentage), and continuous data as mean (standard deviation) or median (Q_1_, Q_3_) if not uniformly distributed. Categorical variables were compared using Chi-square or Fisher exact tests as appropriate. Continuous variables were compared with student T-test or Mann-Whitney U Test. The statistical package used was SPSS version 28, Chicago. Illinois, USA. Statistical significance is p ≤ 0.05. This report complies with the STROBE (Strengthening Reporting of Observational Studies in Epidemiology) recommendation (Von Elm et al., 2007).

### Operational definitions

Wave 1 was for 76 days (June 7, 2020, to Aug 22, 2020).

Wave 2 was for 83 days (Nov 15, 2020, to Feb 6, 2021).

Wave 3 was for 132 days (May 9, 2021, to Sept 18, 2021).

**Asymptomatic infection**: Individuals who tested positive for SARS-CoV-2 using a virologic test (i.e., a nucleic acid amplification test [NAAT] or an antigen test) with no symptoms consistent with Covid-19.

**Mild illness**: Individuals with various symptoms and signs of Covid-19 (fever, cough, sore throat, malaise, headache, muscle pain, nausea, vomiting, diarrhoea, loss of taste and smell, among others) but without dyspnoea or abnormal chest imaging.

**Moderate illness**: Individuals who showed evidence of lower respiratory disease during clinical assessment or imaging and had oxygen saturation measured by pulse oximetry (SpO2) < 94% on room air.

**Severe illness**: Individuals who have SpO2 <94% on room air in addition to a ratio of arterial partial pressure of oxygen to fraction of inspired oxygen (PaO2/FiO2) <300 mm Hg or lung infiltrates >50% of lung fields on imaging.

**Critical illness**: Individuals with any one or more respiratory failure requiring mechanical ventilation, shock, or acute kidney injury requiring haemodialysis.

## Results

### Demographics

A total of 371 patients were admitted across all three waves, with a female preponderance of 61.2% (n=227). There were multiple admissions in 7 patients; 4 had separate admissions in the first and second waves, while 3 had admissions in the first and third waves.

The mean age of all patients is 52.2 ± 16.3, with a range of 12-92 years. The mean age and (range) for the three waves are 52±15 (17-87) years, 53±17 (13-93) years and 48±23 (12-86) years for waves 1, 2 and 3, respectively. Table 1 shows that the patients were predominantly middle-aged and almost all black Africans. The vast majority of patients were referrals from public health facilities. In all three waves, hypertension, diabetes and retroviral disease were the main documented comorbidities.

**Table 1:** Demographics and comorbidities of patients

Characteristics	Wave 1(n=174*)*	Wave 2 (n=168)	Wave 3 (n=29)
**Age group in years**			
13-30	7.4(n=13)	10.1 (n=17)	25 (n=7)
31-50	39.1(n=68)	30.4 (n=51)	21.4 (n=6)
51-65	33.9 (n=59)	35.7 (n=60)	32.1(n=9)
>65	19.5 (n=34)	23.8 (n=40)	21.5 (n=7)
**Females**	66.7 (n=116)	56 (n=94)	58.6 (n=17)
**Referring Centre**			
	92.0 (n=160)	90.5 (n=152)	89.7 (n=26)
Public	8.0 (n=14)	9.5 (n=16)	10.3 (n=3)
Private			
**Ethnicity**	95.4 (n=166)	100.0 (n=168)	100.0 (n=29)
African	4.6 (n=8)	0	0.0
Other			
**Comorbidities**	44.3 (n=77)	28.0 (n=47)	20.7 (n=6)
Diabetes*	52.3 (n=91)	44.0 (n=74)	34.5 (n=10)
Hypertension	23.6 (n=41)	11.9 (n=20)	6.9 (n=2)
Retroviral disease*	4.0 (n=7)	3.0 (n=5)	0.0
TB Asthma/COPD	4.0 (n=7)	4.8 (n=8)	10.3 (n=3)
	4.6 (n=8)	5.4 (n=9)	3.4 (n=1)
Cardiac Disease	5.2 (n=9)	3.0 (n=5)	3.4 (n=1)
CKD			

N is the number of patients per category. TB: Tuberculosis, COPD: chronic obstructive airway disease, CKD: Chronic Kidney Disease.

* ; p<0.05.

### The severity of disease and management strategies involved.

Table 2 shows that most admissions for Covid-19 across all three waves were for moderate disease. Similar proportions of patients across all three waves used steroids, high flow oxygen, ventilator support and ionotropes.

**Table 2:** Severity and management strategies across the three waves of covid 19 in NMAH.

Characteristic	Wave 1 (n=174)	Wave 2 (n=168)	Wave 3 (n=29)
**Severity of disease Moderate**	67.2 (n=117)	67.9 (n=114)	72.4 (n=21)
**Severe**	22.4 (n=39)	17.9 (n=30)	17.2 (n=5)
**Critical**	10.3 (n=18)	14.3 (n=24)	10.3 (n=3)
**Management**			
**strategies**	60.9 (n=106)	66.7 (n=112)	72.4 (n=21)
**Steroid therapy**			
**High flow oxygen**	20.1 (n=35)	15.5 (n=26)	17.2 (n=5)
**Ventilator support**	10.3 (n=18)	14.3 (n=24)	10.3 (n=3)
**Ionotropic support**	5.2 (n=9)	2.4 (n=4)	3.4 (n=1)

N is the number of patients per category.

### Outcomes of covid 19 patients in the three waves.

Table 3 shows a median duration of admission of 7 to 9 days across the 3 waves. The overall mortality across all 3 waves was 31.6%.

**Table 3:** Outcomes of patients admitted with covid-19 across the three waves.

Variable	Wave 1 (n=174)	Wave 2 (n=164)	Wave 3(n=29)
Length of hospital stay in days	7±6	8±7	9±7
Mortality	29.3 (n=51)	31.5 (n=53)	37.9 (n=11)
			

## Discussion

The main findings of this study are that Covid-19 admissions in Nelson Mandela Academic Hospital, Mthatha, Eastern Cape are almost all middle-aged black Africans. The common comorbidities were diabetes, hypertension and HIV. The mortality rate across the three waves ranged from 29.3 to 37.9%. The finding that patients were almost all black Africans is likely a reflection of the population demographics served by the hospital.

Our patients, irrespective of the wave, were mainly females, which is similar to that reported from another study conducted in a regional hospital within Mthatha (Kaswa *et al.*, 2021). Our findings, differ from a pooled analysis of several studies conducted mainly in China with few contributions from Japan, Korea, Italy, the UK and Africa, which report a male dominance in Covid-19 infections (Abate *et al.*, 2020). It is suggested that the higher risk of infectivity in males may be related to increased expression of Angiotensin Converting Enzyme in males, which enhances viral entry into cells in males more than in females (Bwore, 2020). The greater proportions of females in our cases may be related to the gender distribution of the South African population, with more females than males (Statistics South Africa, 2020). Another possibility is that females are more likely than males to seek medical assistance in the event of illness (Goldas *et al.*, 2005).

The mean age of our patients is similar to that reported in another study in Mthatha (Kaswa *et al.*, 2021). The younger age of patients seen during the third wave concurs with that reported by Netcare a South African private hospital group (Maslo *et al.*, 2022). Reasons that have been postulated include a disproportionately high representation of elderly patients among the dead from earlier waves of Covid-19 infection (Maslo *et al.*, 2022). The prioritization of the elderly for vaccination may have contributed to their reduced infections in the third wave. The Omicron variant is highly infectious with the tendency to cause mild disease in young people than the earlier variants (Liu and Rocklar 2022).

The relatively low number of patients admitted during the third wave compared with the two previous waves may be related to several factors, including the tendency of the dominant omicron variant of the Sars-Cov-2 virus to cause mild disease (Liu and Rocklar 2022). Furthermore, there was the availability of vaccination prior to the third wave of the pandemic. The two private hospitals in Mthatha, along with the secondary public hospitals located in the hospital drainage areas, were now admitting patients in their facilities, with secondary public hospitals only referring patients to Nelson Mandela Academic Hospital if there was need to escalate oxygen delivery to modalities including high flow and ventilation or presence of other severe medical illness regardless of the need for oxygen therapy. Hypertension, diabetes and HIV are common comorbidities in our patients, as reported in several other studies (Sanyaolu *et al.*, 2020; Cabore *et al.*, 2022). Our patients with diabetes are likely to be Type 2, considering that most were older than 30 years.

The wide range in our duration of hospital stay is similar to that reported in another study (Rees *et al.*, 2020). Patients who stayed for 1 day in our covid-19 ward were critically ill and died shortly after admission, while extended stays were typically in patients who required prolonged respiratory support. Our mortality rates of 29.3%, 31.5% and 37.9% in waves 1, 2, and 3, respectively, are consistent with Eastern Cape province case fatality of 32.6% (Jassat *et al.*, 2021).

The study’s limitations include the retrospective design, as data were not always available for variable of interest. Obesity is associated with adverse covid-19 outcomes (Poly *et al.*, 2021). However, we were unable to explore obesity in our patients as there were no data on weight and height in the files. Vitamin D deficiency is also associated with adverse Covid-19 outcomes (Mariana, 2021); though all our patients received a stat 50,000 oral dose of calciferol, there was no determination of vitamin D levels in the patients. We could only assume that the patient was infected with the major circulating viral variant per wave, as specific variant identification was not made. It is also plausible that the same patient may have been infected with more than one strain of the virus simultaneously. Our study from a resource constrained rural setting extends on the findings from the nearby Mthatha Regional Hospital which was limited to the first wave and had no data on oxygen therapy.

Conclusions: Our patients from the rural Eastern Cape of South Africa were predominantly females, with a mean mortality of 31.6% across the three waves. Diabetes, hypertension and HIV were the most common comorbidities.
